# Sella turcica dimensions in patients with severe skeletal malocclusion using cone-beam computed tomography: a retrospective observational study

**DOI:** 10.1186/s12903-026-09595-7

**Published:** 2026-08-14

**Authors:** Wenwen Yu, Chunxiang Zhang, Chen Lin, Linkun Zhang, Wensheng Ma

**Affiliations:** 1 Department of Orthodontics, Tianjin Stomatological Hospital, School of Medicine, Nankai University, No. 75, Dagu North Road, Heping District, Tianjin City, 300041 People’s Republic Of China; 2 Tianjin Key Laboratory of Oral and Maxillofacial Function Reconstruction, No. 75, Dagu North Road, Heping District, Tianjin City, 300041 China

**Keywords:** Sella turcica, Sella turcica bridge, Severe skeletal malocclusion, Imaging marker

## Abstract

**Background:**

As traditional two-dimensional cephalometric analyses of skeletal malocclusions often fail to capture complex craniofacial variations. Recent advances in three-dimensional (3D) imaging have highlighted the correlation between sella turcica morphology and skeletal dysmorphology. This study aimed to explore sella turcica dimensions associated with severe skeletal malocclusion using cone-beam computed tomography (CBCT) to explore other imaging features for such patients.

**Methods:**

A total of 240 patients were divided into six groups: normal, skeletal Class III, skeletal Class II, open bite, mandibular deviation, and hypodontia. The sella turcica was reconstructed three-dimensionally from CBCT images using Mimic software, and 13 sella turcica dimensions were measured. Additional cephalometric measurements were performed. For intergroup comparisons of sella turcica dimensions, one-way ANOVA, Kruskal-Wallis test, or Welch’s ANOVA was applied depending on Shapiro-Wilk test and Levene’s test. The post-hoc multiple comparisons between the study groups and the normal group were conducted using Dunnett’s test, the Bonferroni correction, or the Games-Howell test, corresponding to the primary analysis method used. Differences between genders were analyzed using the independent samples t-test or the Mann-Whitney U test, as appropriate. The Pearson’s or Spearman’s correlation tests to assess the relationships between sella turcica dimensions and cephalometric measurements. The prevalence of sella turcica bridging (STB) was compared among groups using the chi-square test.

**Results:**

Sex-based differences were observed in sella turcica dimensions among patients with severe skeletal malocclusion, and females showed significantly lower anterior clinoid distance (ACD), higher sella diameter (SDi) and sella depth (SDe) than males. The mandibular deviation group had a significantly lower dorsum sellae height (DSH) and SDe. The hypodontia group had a lower anterior clinoid distance (ACD) and left sella length (LSL). Notably, female patients with hypodontia demonstrated a more reduction in ACD. No significant differences or strong effect sizes were found for the other sella turcica dimensions among the remaining groups. Significant correlations were identified between sella turcica dimensions (ACD, LSL, posterior right clinoid height [PRCH], DSH and sella length [SL]) and cephalometric measurements (∠Ba-S-N, ∠ANB, ∠NA-PA, pogonion deviation distance [PDD]). STB prevalence was higher in the hypodontia group.

**Conclusions:**

Distinct morphological patterns were associated with specific malocclusion types. The imaging characteristics of the sella turcica may serve as potential exploratory morphological reference for these patients, particularly in mandibular deviation and hypodontia.

**Supplementary Information:**

The online version contains supplementary material available at https://doi.org/10.1186/s12903-026-09595-7.

## Background

The differential diagnosis between mild and severe skeletal malocclusions poses a significant clinical challenge, often resulting in overtreatment or delayed interventions. Severe skeletal malocclusions are characterized by distinctive craniofacial growth patterns that deviate from normal developmental trajectories [1]. Current orthodontic research and imaging protocols predominantly rely on traditional cephalometric analyses of the maxillomandibular complex, cervical vertebrae, and cranial base. However, these conventional parameters fail to address the diagnostic ambiguities inherent in cases of borderline or complex malocclusion. This critical gap underscores the need for novel, anatomically grounded imaging biomarkers to improve diagnostic precision.

In pursuit of improved clinical diagnostic methods for orthodontics, increasing research attention has shifted toward the sella turcica—a saddle-shaped structure located at the cranial base that primarily houses the pituitary gland. Its anatomical characteristics are critical for understanding the craniofacial skeletal structure and function. In imaging, the central point of the sella turcica can be precisely measured using lateral cephalometric radiography or CBCT. Studies have revealed that the anterior portion of the sella turcica originates from cranial neural crest cells, whereas the posterior portion develops from the paraxial mesoderm, depending on the notochord [2, 3]. Furthermore, the morphology of the sella turcica stabilizes after approximately 12 years [2], although certain linear dimensions may continue to increase with age, making it a potential auxiliary candidate for orthodontic diagnosis.

The sella turcica and craniofacial bones share a common embryonic origin, suggesting a potential relationship between their developmental changes. Bastir et al. [4, 5] demonstrated the morphological integration between the facial skeleton and the cranial base through geometric morphometric analyses, indicating that shape variations in the middle cranial fossa—particularly the sella region—are associated with midfacial morphology. Previous studies have consistently demonstrated a close correlation between sella turcica morphology and craniofacial bone development. Cleft lip and palate (CLP) is the second most common facial deformity, and studies have reported that an abnormal sella turcica morphology is present in 86.96% of patients [6]. Recent meta-analyses have highlighted correlations between CLP and sella turcica features: patients with CLP exhibit reduced SDe and SDi, along with abnormal morphologies such as flattening and irregular posterior walls [7]. Sella turcica bridges (STBs) occur in 22.82% patients with CLP, and CLP cases with STB demonstrate a higher prevalence of skeletal Class III malocclusion and dental anomalies (e.g., impacted canines, congenitally missing teeth, and supernumerary teeth) [8]. STB has also been linked to skeletal Class III malocclusion [9, 10] and dental developmental anomalies (including impacted canines, tooth transpositions, and third molar agenesis) [11]. Therefore, the morphology of the sella turcica is closely related to the development of the facial skeleton and teeth. However, current research findings are inconsistent because of differences in measurement methods, imaging modalities, and classification criteria. Consequently, if the morphology of the sella turcica is to be used as a potential auxiliary diagnostic indicator for evaluating the malocclusion, more accurate imaging tools and refined, standardized measurement methods are required.

Traditional lateral cephalograms—two-dimensional (2D) images prone to projection errors, magnification, overlapping anatomical structures, and head rotation—remain widely used but lack precision [12]. By contrast, CBCT offers high-resolution 3D imaging, clearer visualization of complex anatomy, shorter acquisition time, lower radiation exposure, and greater safety for pediatric and adolescent applications [13, 14]. Even so, existing CBCT-based studies examining correlations between the sella turcica and malocclusion are limited by small sample sizes, inconsistent measurement protocols, and suboptimal imaging accuracy. To address these limitations, this retrospective study analyzed the morphology of the sella turcica in patients with severe skeletal malocclusion to identify specific morphological markers. This study provided preliminary, hypothesis-generating evidence for sella turcica morphology as an auxiliary assessment factor for craniofacial skeletal and dental anomalies.

## Methods

### Patient selection

This cross-sectional retrospective study included patients who visited the Tianjin Stomatological Hospital of Nankai University between March 1, 2019, and December 31, 2024. A total of 240 participants (143 females and 97 males; mean age: 21.54 years) were enrolled. This study was approved by the Medical Research Ethics Committee of Tianjin Stomatological Hospital (approval no.: PH2024-Q-014). Written informed consent was obtained from all participants or their legal guardians.

Participants were categorized into six groups according to established diagnostic criteria: normal, skeletal Class III, skeletal Class II, open bite, mandibular deviation, and hypodontia. Eligible patients who met the inclusion criteria were selected consecutively until each group contained 40 participants.

The inclusion criteria were as follows: (a) availability of a clearly captured CBCT image under standard conditions; (b) age ≥ 16 years; (c) no history of orthodontic or orthognathic treatment; (d) availability of complete clinical records, including facial and intraoral photographs, panoramic radiographs, and lateral cephalograms obtained at initial visit; (e) voluntary participation in the study. The exclusion criteria were as follows: (a) presence of syndromic or systemic diseases; (b) history of radiotherapy, chemotherapy, or long-term use of medications affecting skeletal development; and history of (c) orthodontic or orthognathic treatment, (d) dental trauma, (d) head and neck surgery, and (e) cleft lip and/or palate.

#### Group definitions

Participants were divided into one normal (control) and five study groups: skeletal Class III, skeletal Class II, open bite, mandibular deviation, and hypodontia.

- Normal group: patients diagnosed with a skeletal Class I relationship, average vertical pattern, and normal occlusion without missing teeth.
- Hypodontia group: congenital absence of three or more permanent teeth.
- Skeletal Class III, Class II, open bite, and mandibular deviation groups: patients meeting the criteria for orthodontic–orthognathic treatment, without missing teeth, and exhibiting minimal growth potential (post-peak growth period).

Additional diagnostic criteria of these four groups were as follows:

- Skeletal Class III with (i) an A (subspinale) point–Nasion–B (supramental) point angle (∠ANB) of <-4° and (ii) a lower incisor to mandibular plane angle (∠L1-MP) of < 83° [15].
- Skeletal Class II with (i) ∠ANB ≥ 6° or a profile angle of < 160° [16] and (ii) notable mandibular retrusion.
- Open bite with (i) an overbite depth indicator (ODI) of < 74–75°, (ii) a vertical open bite distance (OBD) of ≥ 4 mm between upper and lower incisors, and (iii) a diagnosis of severe skeletal open bite requiring orthodontic–orthognathic treatment [17].
- Mandibular deviation with (i) a pogonion deviation distance (PDD) from the facial midline of > 4 mm in both postural and intercuspal positions [18] and (ii) compensatory dental changes in both arches.

All patient screenings and classifications were confirmed by two senior attending physicians according to the above criteria.

### Imaging examination and measurements

Panoramic and lateral cephalometric radiographs were obtained for all participants using the same ORTHOPHOS XG5 unit (Sirona, Germany) under standardized conditions. Imaging parameters were set to 64 kV and a maximum current of 8 mA. All raw images were saved in JPG format and analyzed using Dolphin Imaging software (version 11.9.7.24) for lateral cephalometric measurements. In addition to the grouping parameters (∠ANB, angle between the Nasion–A point line and the A-Po (pogonion) line [∠NA-PA], ∠L1-MP, and ODI), the following cranial measurements—adapted from Proff [19] and Canigur [20]—were performed to evaluate the correlation between sella turcica morphology and craniofacial dimensions: anterior cranial base (S-N, mm), anterior cranial base angle (∠FH-SN, °), posterior cranial base (S-Ba, mm), total cranial base (N-Ba, mm), total cranial base angle (∠Ba-S-N, °), upper face height (N-anterior nasal spine [ANS], mm), lower face height (ANS-Me, mm), anterior face height (Na-Me, mm), posterior facial height (S-Go, mm), total face height (N-Gn, mm), maxillary length (ANS-posterior nasal spine [PNS], mm), mandibular angle (∠Co-Go-Me, °), mandibular ramus height (Co-Go, mm), mandibular body length (Go-Gn, mm), mandibular plane angle (∠SN-GoGn, °), and Frankfort–mandibular plane angle (FMA; ∠MP-FH, °), and Wits value.

CBCT scans were acquired using a KaVo 3D eXam unit (Imaging Science, USA) under consistent protocols. The scanning parameters included a voxel size of 0.3 mm, field of view of 23 × 17 cm, voltage of 120 kV, and tube current of 37.1 mA.

### Measurement of sella turcica parameters

Sella turcica measurements were performed following previously established methodologies [21, 22]. A total of 13 measurements were designed to comprehensively evaluate sella turcica dimensions:

1. Anterior clinoid distance (ACD): distance between the left and right anterior clinoid processes (ACP).
2. Posterior clinoid distance (PCD): distance between the left and right posterior clinoid processes (PCP).
3. LSL: distance between the left ACP and PCP.
4. RSL: distance between the right ACP and PCP.
5. Anterior right clinoid height (ARCH): vertical distance from the right ACP to the Frankfort horizontal′ (FH′) plane.
6. Posterior right clinoid height (PRCH): vertical distance from the right PCP to the FH′ plane.
7. Anterior left clinoid height (ALCH): vertical distance from the left ACP to the FH′ plane.
8. Posterior left clinoid height (PLCH): vertical distance from the left PCP to the FH′ plane.
9. TSH: vertical distance from the turberculum sellae (TS) to the FH′ plane.
10. DSH: vertical distance from the dorsum sellae point (DS) to the FH′ plane.
11. SL: distance between TS and DS in the midsagittal plane.
12. SDi: distance from TS to the most posterior point of the posterior wall of the sella turcica in the mid-sagittal plane.
13. SDe: perpendicular distance from the sella floor to the line connecting TS and DS in the mid-sagittal plane.

Here, the FH′ plane refers to the FH plane translated to pass through the sella floor point.

All CBCT images were reoriented using Mimics software (version 21.0) based on the horizontal plane (defined by the bilateral orbitale and right porion) and the midsagittal plane (perpendicular to the horizontal plane, passing through the nasion and basion). A 3D reconstruction of the sella region was performed, followed by landmark identification and measurements.

STB was evaluated according to the classification proposed by Ortiz [23], which objectively quantifies the degree of calcification. Participants were divided into two groups based on the ratio between the ACP–PCP distance and the TS–DS length: no bridging (ratio: ≥60%) and presence of bridging (ratio: <60%).

### Sample size calculation and reliability analysis

Sample size estimation was performed using PASS software (version 15.0). Based on the retrospective nature of this study and preliminary findings, parameters were set to an α-value of 0.05 and a statistical power of 80%. The calculated minimum sample size was 37 participants per group. Accordingly, 40 participants were enrolled in each group, yielding a total sample size of 240 participants across the six groups.

To assess measurement reliability, 100 randomly selected samples were re-measured by the same investigator after a 2-week interval. Intraclass correlation coefficients (ICCs) were calculated to evaluate consistency between the two measurement sessions.

### Statistical analysis

Data analyses were performed using SPSS Statistics (version 27.0). The normality and homogeneity of variance were assessed for all variables. Continuous data are presented as means ± standard or median with interquartile range, based on their distribution deviation. Categorical variables were presented as numbers and percentages. The following method were applied to compare ∠ANB, Wits, ∠NA-PA, ∠L1-MP, ODI, age, and sella turcica dimensions among the groups. For continuous variables that satisfied both normality (assessed using the Shapiro-Wilk test) and homogeneity of variances (assessed using Levene’s test), intergroup comparisons were conducted using one-way analysis of variance (ANOVA). If a significant overall difference was detected, post-hoc multiple comparisons between the study groups and the normal group were performed using Dunnett’s test. For data that followed a normal distribution but violated the assumption of homogeneity of variances, Welch’s ANOVA was employed for the overall comparison, followed by the Games-Howell test for post-hoc analysis against the normal group. For continuous variables that did not follow a normal distribution, the non-parametric Kruskal-Wallis test was used. When the overall test was significant, post-hoc pairwise comparisons were conducted with a Bonferroni correction. Differences between genders for continuous variables were analyzed using the independent samples t-test or the Mann-Whitney U test, as appropriate, depending on whether the assumptions of normality and variance homogeneity were met.

Correlations between sella turcica dimensions and cranial base parameters were evaluated using Pearson’s test for normally distributed data and Spearman’s test for non-normally distributed data. Chi-square tests were used to compare the prevalence of STB between the groups. A *P*-value of < 0.05 was considered statistically significant.

## Results

### Reliability analysis

The ICC values in this study ranged from 0.962 to 0.995, indicating good intra-group consistency and minimal individual measurement error.

### Group classification results

Demographic characteristics, including age and sex distribution, across the groups are summarized in Table 1. Based on the diagnostic criteria for three-dimensional skeletal malocclusion and hypodontia, the participants were categorized into five study groups. The skeletal Class III group exhibited significantly smaller ∠ANB and ∠L1-MP values (-5.05°(-6.08°,-3.83°)and 78.61° ± 8.24°, respectively) compared with the normal group, consistent with severe skeletal Class III characteristics. The skeletal Class II group showed significantly larger ∠ANB and ∠NA-PA values (8.95°(8.13°,10.00°) and 19.79° ± 3.91°, respectively) than the normal group, meeting the diagnostic criteria for severe skeletal Class II malocclusion. The open bite group had an OBD of 4.29 ± 1.58 mm and ODI of 65.76 ± 9.89°; the mandibular deviation group exhibited a PDD of 7.14 ± 2.38 mm; and the hypodontia group presented an average of 5.23 ± 2.27 missing teeth, with all values significantly greater than those in the normal group, confirming the respective severe malocclusion criteria. No significant age differences were observed among these groups. Additionally, data from the mandibular deviation group indicated a tendency toward skeletal Class III malocclusion with − 6.75(-9.63, -2.90) mm of Wits. Sex-based analysis indicated that no differences were found across the groups except ∠L1-MP in skeletal Class II group and years in open bite group (Table S1).

**Table 1:** The cephalometric measurements, open bite distance, pogonion deviation distance, hypodontia, age and sex distribution of every group

	Normal	Skeletal Class III	Skeletal Class II	Open Bite	Mandibular Deviation	Hypodontia	Overall Test	*P*-value
∠ANB(°)	3.10(2.10,4.20)	-5.05(-6.08, -3.83) ^aaa^	8.95(8.13,10.00) ^aaa^	2.55(0.33,4.30)	-0.25(-1.60,1.83) ^aa^	2.90(1.33,5.08)	H (5) = 177.612	<0.001
Wits(mm)	-0.90(-2.00,1.13)	-12.10(-15.00, -10.13)^aaa^	6.15(4.05,7.98)^aaa^	-1.00(-4.98,2.38)	-6.75(-9.63, -2.90)^aaa^	-0.30(-2.63, -1.88)	H (5) = 163.316	<0.001
∠NA-PA(°)	6.98 ± 3.81	-10.19 ± 5.46 ^bbb^	19.79 ± 3.91 ^bbb^	6.46 ± 6.86	0.64 ± 5.37 ^bbb^	4.98 ± 6.79	Welch’s F (5, 108.233) = 172.533	<0.001
∠L1-MP(°)	96.63 ± 5.67	78.61 ± 8.24 ^ccc^	103.16 ± 8.64 ^cc^	91.58 ± 8.53 ^c^	85.51 ± 7.48 ^ccc^	92.14 ± 9.35	F (5,234) = 44.777	<0.001
ODI(°)	71.34 ± 4.54	52.91 ± 7.29 ^bbb^	80.72 ± 8.60 ^bbb^	65.76 ± 9.89 ^b^	83.24 ± 5.42 ^bbb^	73.34 ± 8.38	Welch’s F (5, 107.705) = 98.421	<0.001
OBD(mm)	0	0.21 ± 0.63	0.22 ± 0.61	4.29 ± 1.58	0.06 ± 0.23	0		
PDD(mm)	1.00 ± 1.05	1.68 ± 1.72	0.76 ± 0.94	1.29 ± 1.41	7.14 ± 2.38	1.02 ± 1.58		
MT (n)	0	0	0	0	0	5.23 ± 2.27		
Age (year)	22.00(17.25,27.00)	19.00(17.25,23.50)	2 1.00(18.00,26.50)	19.00(17.25,23.00)	19.00(17.00,21.00)	21.00(18.00,26.00)	H (5) = 8.671	0.123
Male (n)	18	18	15	15	15	16		
Female (n)	22	22	25	25	25	24		

*ODI* Overbite Depth Indicator, *OBD* Open Bite Distance, *PDD* Pogonion Deviation Distance, *MT* missing teeth, *n* number of subjects

The test statistics H, Welch’s F and F are derived from the Kruskal-Wallis test, Welch’s ANOVA and one-way ANOVA, respectively. The *P*-value indicates statistical significance for the corresponding test. Significant difference vs. normal (*P* < 0.05) by: a, Bonferroni test; b, Games-Howell test; c, Dunnett’s test. The a, b, c<0.05;aa, bb, cc<0.01;aaa, bbb, ccc<0.001

### Sella turcica dimensions in different severe skeletal malocclusion types

The mean values of the 13 sella turcica dimensions are presented in Table 2. Males had significantly bigger ACD (26.72 ± 2.69 mm) and smaller SDi (10.83 ± 1.35 mm), SDe (7.78 ± 1.31 mm) than females (25.11 ± 2.52 mm, 11.39 ± 1.47 mm, 8.13 ± 1.38 mm, respectively). While the difference of SDi and SDe corresponded to a small-to-medium effect size according to Cohen’s d (-0.394 and − 0.264).

**Table 2:** The mean and standard deviation of sella turcica dimensions in total and sex groups

	Total(*n* = 240)	Male(*n* = 97)	Female(*n* = 143)	*P*-value	Cohen’s d (95% CI)
ACD	25.76 ± 2.71	26.72 ± 2.69	25.11 ± 2.52	<0.001	0.624(0.359, 0.887)
PCD	16.76(15.06,18.66)	17.30(15.00,19.33)	16.50(15.05,18.07)	0.080	0.344(-0.296, 0.979)
LSL	7.27(5.60,8.74)	7.32(5.57,9.03)	7.23(5.70,8.40)	0.544	0.157(-0.290, 0.959)
RSL	6.69 ± 2.28	6.71 ± 2.45	6.69 ± 2.15	0.946	0.009(-0.249, 0.267)
ARCH	7.53 ± 1.61	7.53 ± 1.51	7.53 ± 1.69	0.996	0.001(-0.257, 0.258)
PRCH	8.49 ± 1.76	8.49 ± 1.78	8.49 ± 1.75	1.000	0.000(-0.258, 0.258)
ALCH	7.38 ± 1.71	7.39 ± 1.53	7.37 ± 1.82	0.910	0.015(-0.243, 0.273)
PLCH	7.99 ± 1.94	7.93 ± 1.94	8.04 ± 1.95	0.658	-0.058(-0.316, 0.200)
TSH	8.16 ± 1.46	7.98 ± 1.35	8.29 ± 1.52	0.102	-0.216(-0.474, 0.043)
DSH	7.79 ± 1.76	7.58 ± 1.72	7.93 ± 1.78	0.130	-0.200(-0.458, 0.059)
SL	10.26 ± 1.54	10.19 ± 1.53	10.29 ± 1.54	0.623	-0.065(-0.323, 0.193)
SDi	11.16 ± 1.45	10.83 ± 1.35	11.39 ± 1.47	0.003	-0.394(-0.653, -0.133)
SDe	7.99 ± 1.36	7.78 ± 1.31	8.13 ± 1.38	0.046	-0.264(-0.523, 0.005)

The *P*-value and Cohen’s d (95% confidence interval) were obtained from independent samples t-tests or Mann-Whitney U tests

Comparative analysis between each study group and the normal group revealed that mandibular deviation group showed a significantly lower DSH (7.11 ± 1.79 mm vs. 8.10 ± 1.67 mm) and SDe (7.49 ± 1.24 mm vs. 8.26 ± 1.43 mm), while the hypodontia group had significantly smaller ACD (24.72 ± 2.77 mm vs. 26.26 ± 2.30 mm) and LSL (6.20(4.01,7.66) mm vs. 7.36(5.93,8.35) mm). All these four significant indicators demonstrated the medium or strong effect sizes (Cohen’s d = 0.452-0.602). No other significant differences in sella dimensions were detected among the groups (Table 3).

**Table 3:** The mean and standard deviation of sella turcica dimensions in six groups

	Normal	Skeletal Class III		Skeletal Class II	Open Bite	Mandibular Deviation	Hypodontia	Overall Test	*P*-value	Cohen’s d (95% CI)
ACD	26.26 ± 2.30	25.72 ± 2.55	25.94 ± 3.04		26.38 ± 2.22	25.55 ± 3.06	24.72 ± 2.77 ^a^	F (5,234) = 1.986	0.081	0.602(0.152,1.049)
PCD	17.18(15.03,18.99)	16.19(15.09,17.6)	16.68(14.98,18.11)		16.60(15.11,18. 59)	16.94(14.54,18.31)	16.91(15.78,19.61)	H (5) = 3.337	0.648	
LSL	7.36(5.93,8.35)	6.78(5.53,9.76)	6.86(5.11,8.08)		7.69(6.22,9.37)	7.79(6.16,9.04)	6.20(4.01,7.66) ^b^	H (5) = 16.232	0.006	0.452(0.007,0.895)
RSL	6.57 ± 2.56	6.90 ± 2.39	6.39 ± 2.09		7.44 ± 2.02	6.86 ± 2.36	6.02 ± 2.06	F (5,234) = 1.864	0.101	
ARCH	7.79 ± 1.78	7.25 ± 1.98	7.52 ± 1.49		7.64 ± 1.29	7.27 ± 1.56	7.72 ± 1.51	F (5,234) = 0.788	0.560	
PRCH	8.52 ± 1.71	8.15 ± 1.83	8.94 ± 1.98		8.42 ± 1.48	8.10 ± 1.75	8.82 ± 1.71	F (5,234) = 1.547	0.176	
ALCH	7.41 ± 1.39	7.11 ± 2.04	7.23 ± 1.79		7.79 ± 1.50	7.04 ± 1.66	7.71 ± 1.75	F (5,234) = 1.358	0.241	
PLCH	8.19 ± 2.22	7.48 ± 1.86	7.92 ± 2.12		8.24 ± 1.94	7.94 ± 1.69	8.22 ± 1.78	F (5,234) = 0.907	0.477	
TSH	8.37 ± 1.56	7.89 ± 1.74	8.06 ± 1.49		8.41 ± 1.05	8.02 ± 1.47	8.23 ± 1.39	F (5,234) = 0.789	0.558	
DSH	8.10 ± 1.67	7.43 ± 2.06	8.23 ± 1.86		7.89 ± 1.55	7.11 ± 1.79 ^a^	7.96 ± 1.41	F (5,234) = 2.426	0.036	0.571 (0.121,1.015)
SL	10.25 ± 1.48	10.96 ± 1.76	9.87 ± 1.48		10.29 ± 1.28	10.32 ± 1.44	9.80 ± 1.54	F (5,234) = 3.055	0.011	
SDi	11.35 ± 1.28	11.41 ± 1.61	10.84 ± 1.79		11.15 ± 1.28	11.03 ± 1.41	11.18 ± 1.24	F (5,234) = 0.82	0.536	
SDe	8.26 ± 1.43	7.58 ± 1.65	8.23 ± 1.35		8.15 ± 1.09	7.49 ± 1.24 ^c^	8.21 ± 1.16	Welch’s F (5, 108.907) = 2.683	0.025	0.572(0.122,1.016)

The test statistics F, H, and Welch’s F are derived from the one-way ANOVA, Kruskal-Wallis test, and Welch’s ANOVA, respectively. The *P*-value indicates statistical significance for the corresponding test. Significant difference vs. normal (*P* < 0.05) by: a, Dunnett’s test; b, Bonferroni test; c, Games-Howell test. The a, b, c<0.05;aa, bb, cc<0.01;aaa, bbb, ccc<0.001.Cohen’s d (95% CI) is provided where post-hoc tests indicated a significant difference from the control

Further sex-stratified analyses indicated that, there were no differences between each study group and normal in male (Table S2). But in female, the hypodontia group exhibited a smaller ACD (24.04 ± 2.54 mm) than those in the normal group (25.88 ± 2.26 mm) (Table S3). Additionally, despite the lack of significant differences between the normal group and PCD in skeletal Class III, and SDe in mandibular deviation, and LSL and RSL in open bite group, which exhibited medium-to-strong effect sizes (Cohen’s d = 0.657, 0.717, -0.573, -0.537 respectively).

### Correlations between sella turcica dimensions and cranial parameters

**Fig. 1:**
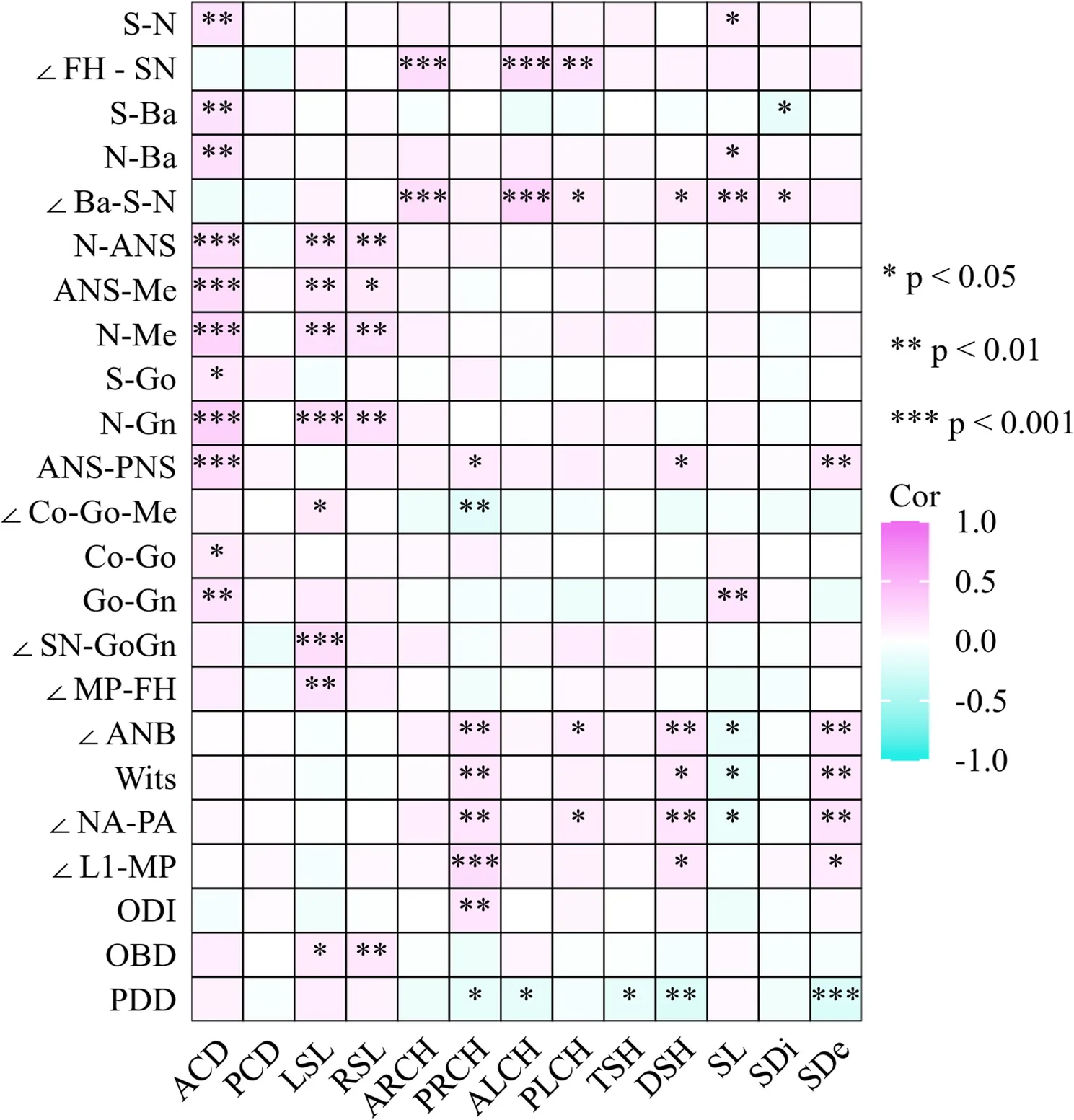
Correlation between sella turcica dimensions and cephalometric measurements. Heatmap shows the correlations between 13 sella turcica measurements and multiple cephalometric measurements related to the cranial base, maxilla, and mandible. Pink indicates a positive correlation, whereas blue represents a negative correlation. The darker the color, the stronger the correlation; conversely, the weaker the correlation, the lighter the color

Correlation analysis was conducted to explore the associations between sella turcica dimensions and other cranial parameters (Fig. 1). Multiple significant correlations were identified. Among the 13 sella parameters, ACD showed the most correlations: positive associations with cranial base measurements (S-N, S-Ba, and N-Ba), maxillary length (ANS-PNS), mandibular body length (Go-Gn), and vertical skeletal parameters (N-ANS, ANS-Me, N-Me, S-Go, N-Gn, Co-Go). LSL was the second most correlated measure, showing positive relationships with N-ANS, ANS-Me, N-Me, N-Gn, ∠Co-Go-Me, ∠SN-GoGn, ∠MP-FH, and OBD—mostly related to vertical development. RSL was positively correlated with N-ANS, ANS-Me, N-Me, N-Gn, and OBD, which was consistent with LSL. PRCH was also the second correlated parameter, which was positively associated with ANS-PNS, ∠ANB, Wits, ∠NA-PA, ∠L1-MP, and ODI but negatively associated with ∠Co-Go-Me and PDD. DSH and SDe exhibited similar correlation patterns with the cranial parameters as PRCH, except for ∠Co-Go-Me, ∠Ba-S-N and ODI. ARCH was positively correlated with cranial base parameters (∠FH-SN and ∠Ba-S-N); ALCH was similar to ARCH but in addition negatively with PDD; PLCH was positively correlated with ∠FH-SN, ∠Ba-S-N, ∠ANB, and ∠NA-PA; SL was positively correlated with S-N, N-Ba, ∠Ba-S-N, and Go-Gn but negatively with ∠ANB, Wits, ∠NA-PA; PCD showed no statistical differences with every cranial parameter; TSH was negatively correlated only with PDD; and SDi was negative with S-Ba and positive with ∠Ba-S-N.

Cranial parameters were ranked by the number of correlations with sella dimensions in the following order: ∠Ba-S-N, ∠ANB, ∠NA-PA, PDD, ANS-PNS, Wits, ∠FH-SN, N-ANS, ANS-Me, N-Me, N-Gn, ∠L1-MP, S-N, S-Ba, N-Ba, ∠Co-Go-Me, Go-Gn, OBD, S-Go, Co-Go, ∠SN-GoGn, ∠MP-FH, and ODI. PDD was negatively correlated with sella turcica paramters.

Notably, these cranial parameters showed only weak correlations with sella turcica measurements.

### Prevalence of STB

The prevalence of STB in the control group was 25% (Table 4). Among the study groups, the prevalence rates were 35% for skeletal Class III malocclusion, 37.5% for skeletal Class II malocclusion, 27.5% for open bite, 32.5% for mandibular deviation, and 47.5% for hypodontia. All groups had a higher prevalence of STB than the normal group, with only the hypodontia group reaching statistical significance. However, after Bonferroni correction, no significant differences of STB were observed among all groups. No significant difference in STB prevalence was observed between males and females.

**Table 4:** The difference of STB in six groups and genders

	STB^+^		STB^−^		χ²	*P*-value	Bonferroni-corrected *P*-value
	*n*	%	*n*	%			
Normal	10	25.00	30	75.00			0.01
Skeletal Class III	14	35.00	26	65.00	0.952	0.329	
Skeletal Class II	15	37.50	25	62.50	1.455	0.228	
Open Bite	11	27.50	29	72.50	0.065	0.799	
Mandibular Deviation	13	32.50	27	67.50	0.549	0.459	
Hypodontia	19	47.50	21	52.50	4.381	0.036	
Total	81	33.75	159	66.25			
Male	31	31.96	66	68.04	0.234	0.629	
Female	50	34.97	93	65.03			

*STB* sella turcica bridging, *n* number of subjects

Statistical significance was assessed by chi-square tests, with *P*-values reported

## Discussion

The sella turcica maintains a close anatomical relationship with surrounding craniofacial structures. It is bordered anteriorly by the frontal bone, posteriorly by the occipital bone, and laterally by the temporal and sphenoid bones. Owing to its strategic position, the sella turcica serves as a critical junction between the brain and the endocrine system. Pathologies or injuries in this region can impair pituitary function, disrupt endocrine homeostasis, and consequently affect general growth and craniofacial bone development [24]. Conventional two-dimensional lateral cephalograms are prone to radiographic distortions, which have historically limited detailed investigations into the morphology of the sella turcica. In the present study, we employed CBCT-based three-dimensional reconstruction and a systematic measurement protocol to conduct a comprehensive analysis of sella turcica morphology in patients with severe skeletal malocclusions across multiple planes—sagittal (skeletal Class III and II), vertical (open bite), coronal (mandibular deviation), and dental (hypodontia). Our results demonstrated significant correlations between sella turcica morphology and different types of severe skeletal malocclusion, suggesting its relation as an auxiliary imaging-based diagnostic indicator for assessing severe malocclusions.

As mentioned previously, the morphology of the sella turcica became essentially stable by 12 years of age [2]. Tepedino [25] further confirmed above view, which the sella turcica is significantly larger at the age of 13 years without altered shape. Given that craniofacial growth in male adolescents and sella turcica maturity typically plateaus after 16 years of age according previous study [21]. We selected this age group to minimize potential measurement variance from ongoing development. Reported sella turcica dimensions vary by population. As the Asian descent, Yemeni populations exhibit similar sella turcica dimensions with ours [26]. Conversely, Saudi populations show significantly larger dimensions (length: 11.57 ± 4.73 mm; diameter: 11.54 ± 6.13 mm; depth: 9.54 ± 5.23 mm) [27] than our results. Additionally, Saudi populations showed sex-based differences in length but not in diameter or depth, which is in contrast to our findings. Our SL and SDi values are generally consistent with those reported by Chou [22], although our ACD, PCD, LSL, and RSL values were slightly higher in both sexes. This variance may be attributed to regional as Chou’s study focused on a Taiwanese population with a broad age range (18–40 years). Comparisons with Marya’s study [28] revealed considerable differences: their SL, SDi, and SDe values in males with Angle’s Class I, II, and III malocclusions were generally larger than ours, whereas females with Class II and III malocclusions had significantly smaller SL values. These disparities likely arise from ethnic variations and different patient selection criteria. Notably, our study found that SDi and SDe were significantly greater in females, a finding consistent with that of Nabavizadeh [29], who reported a larger depth and diameter in females using 2D cephalometry. Muhammed [30] identified race, age, and sex as significant factors influencing SDi. Collectively, these findings indicate that the imaging equipment, geographic origin, ethnicity, age, and measurement techniques can contribute to variations in sella turcica metrics.

Several studies have reported associations between sella turcica morphology and sagittal craniofacial relationships (Angle’s Classes I, II, and III). Using lateral cephalograms, Issrani [27] and Shrestha [31] found that patients with Class III malocclusion had significantly greater SL, SDe and SDi values. Önal [32] suggested that Class III malocclusion is associated with greater SL, whereas Class II malocclusion is associated with greater SDe. Marya [28] reported strong positive correlations among SL, SDi, and SDe in patients with skeletal Class II malocclusion. Our results did not show significant changes of sella turcica dimensions in skeletal Class II and III malocclusions. In the study of Chou [22], using CBCT measurements, they also found no correlation among sella dimensions (ACD, PCD, LSL, RSL, SL, and SDi), STB prevalence, and different craniofacial skeletal types (Angle’s Class I, II, and III). Silveira [33] supported our and Chou’s conclusion that the sella turcica size is unrelated to cranial morphology in patients with Class II and III malocclusion.

Vertical skeletal variations have also been associated with sella turcica dimensions. A higher prevalence of STB (51.1%) was observed in patients with a low mandibular plane angle [34]. In Yan’s study [21], patients with Angle’s Class I malocclusion were categorized into low-, normal-, and high-angle groups, and their sella turcica was evaluated using CBCT. The results indicated that patients in the low-angle group had greater PCD but reduced PLCH and PRCH, along with a higher STB prevalence. By contrast, our study did not find significant morphological differences in the sella turcica of patients with an open bite. Nabavizadeh [29] reported that the probability of exhibiting an open bite was 8.57 and 1.82 times greater in individuals with STB than in those without. Furthermore, the occurrence of a long-face deformity was negatively correlated with SL and positively correlated with SDe but showed no correlation with SDi. In contrast to these findings, our results demonstrated significant positive correlations between open bite indices (ODI and OBD) and LSL, RSL, PRCH. This discrepancy with the findings of Nabavizadeh may be attributed to their reliance on 2D radiography, where superimposition artifacts could obscure the influence of other sella turcica dimensions on open bite development.

Studies investigating the relationship between sella turcica morphology and mandibular deviation are scarce. This is likely attributed to the challenge of identifying participants with pure skeletal mandibular asymmetry, as such cases often present with confounding developmental anomalies such as skeletal Class III malocclusion (~ 48% of whom have significant asymmetry [35]), open bite, or temporomandibular joint disorders. This is the first study to describe alterations in sella turcica morphology associated with severe jaw deviation. Notably, patients with mandibular deviation had significantly reduced DSH and SDe. Furthermore, the degree of mandibular deviation was significantly negatively correlated with several vertical measurements of the sella turcica. Despite a mere 0.09 mm difference in skeletal Class III SDe compared to the mandibular deviation group, no statistical significance was observed, indicating that an SDe around 7.5 mm could be a potential clinical cutoff for discriminating deviation from crossbite. Further investigations are warranted to fully elucidate the characteristics of the sella turcica in patients with mandibular deviation.

Numerous studies have demonstrated a close relationship between sella turcica morphology and abnormalities in tooth development, including missing and impacted teeth. Kaya [11] evaluated the sella turcica in a large cohort with dental developmental anomalies and found a significantly reduced length and diameter in patients with tooth transpositions. Similarly, Alqahtani [36] reported a significant reduction in SL among patients with a congenital absence of maxillary lateral incisors. Canigur [2] observed a noticeably smaller SDi in patients with bilaterally impacted maxillary canines. Furthermore, the absence of maxillary second premolars and severe tooth agenesis have been associated with general intellectual delay (ID) and an increased prevalence of STB [37]. Our findings presented significantly smaller ACD and LSL in hypodontia patients, especially in females. This result underscored the superiority and importance of CBCT, as these two indicators could only be detected by CBCT imaging.

Despite statistical significance in some sella turcica dimensions, the weak correlation suggested limited utility of sella turcica dimensions alone as a diagnostic indicator, and its direct clinical applicability required further validation.

STB is an anatomical variant characterized by the ossification of the ligament between the anterior and posterior clinoid processes. It is a prenatal developmental anomaly whose size remains stable over time and can occur unilaterally or bilaterally [38]. STB is a common morphological variation of the sella turcica, with a recently reported prevalence of approximately 18% [39]. Notably, significantly higher bridging rates (35.9%) have been documented in Yemeni populations [26]. In the present study, STB prevalence was 33.75%, significantly higher than that reported by Sobouti (~ 18%) [39] and Cuschieri (~ 25%) [40] but lower than that reported by Ortiz (~ 50%) [23] and Alqahtani (~ 47%) [36]. These disparities likely stem from differences in study populations, age ranges, and selection criteria. The higher rate reported in this study may be attributed to our focus on patients with severe skeletal deformities, a population in which STB is consistently reported to be more common, as confirmed by other studies [40]. Our study did not find a significant sex difference in the overall prevalence of STB. However, CBCT-based analysis indicated a higher prevalence of STB on the right side, particularly among females [41]. Using lateral cephalometry in an Iranian population, Sobuti [10] found a significant association between STB and craniofacial skeletal patterns, with a higher prevalence in patients with skeletal Class III malocclusion. This finding has been corroborated by other studie [11, 42–45]. STB is also associated with other dental anomalies, including numeric anomalies (e.g., missing lateral incisors or premolars), root dilaceration, and tooth impaction [37, 46, 47]. It has also been linked to vertebral abnormalities and certain syndromes (e.g., Down, Marfan, or Turner syndrome) [48, 49]. Therefore, clinicians should recognize the anatomical significance of STB during radiographic examinations, as its presence can aid in the comprehensive diagnosis of associated conditions.

STB is one type of sella turcica abnormality. Kucia [50] further categorized the morphologies into 11 types. A hypertrophic posterior clinoid process is significantly more common in patients with skeletal Class III malocclusions [45], as well as the STB, irregular dorsum, and pyramidal dorsum [51]. Utsunomiya [52] analyzed 47 fetal specimens using geometric morphometrics to assess the morphological integration between the facial skeleton and the sella turcica. Their analysis found that the curved anterior wall of the sella turcica was associated with an immature facial morphology, characterized by mandibular prognathism, maxillary retrognathism, and smaller zygomatic bones. By contrast, the posterior wall exhibited less variability. Based on our data, in patients with mandibular deviation, the negative correlation between the degree of PDD and vertical indicators of sella turcica toward a flat shape of sella. Meanwhile, in patients with hypodontia, the smaller ACD and LSL toward an irregular trapezoid when viewed in the transverse section (with the face oriented forward).

Although this study included patient samples representing severe skeletal malocclusion across various dimensions, several limitations must be acknowledged. First, we did not control for vertical dimension criteria. This decision was primarily based on our study population of patients with severe skeletal malocclusion. In such individuals (particularly those with skeletal Class III, Class II, open bite, or mandibular deviation), jaw development deviated from the normal range, inevitably leading to vertical discrepancies. Our strategy introduced potential confounding, such as the possible overlap between different skeletal malocclusion types, which might affect the purity of the results. Future studies are planned to strictly screen participants under unified vertical classifications. Second, this study employed conventional cephalometric parameters for grouping rather than individualized measurements. As the control group consisted of individuals with normal occlusion, and traditional measurements were more universally applied, we utilized this conventional classification system to ensure consistent analytical standards and clinical applicability. Naturally, subsequent research focusing on specific malocclusion types will adopt individualized measurement criteria for in-depth analysis. Third, this study was exploratory in nature, aiming to explore specific sella turcica indicators as potential imaging diagnostic references, which should avoid excessive interpretation and extrapolation. Forth, the clinical relevance of most observed inter-group differences may be limited due to their small effect sizes. Consequently, findings require validation through studies with larger sample sizes. Furthermore, in our results, some sella turcica dimensions in females showed favorable effect sizes yet lacked statistical significance. This suggests that future research needs to expand the sample size to enhance statistical power. Finally, all participants were recruited from the Orthodontic Department of our hospital, which does not represent the general population. Consequently, the results may exhibit regional bias, as patients seeking orthodontic care often differ from the general public in terms of awareness, income, and educational level [53].

## Conclusions

The imaging characteristics of sella turcica morphology are associated with severe malocclusion. Sexual dimorphism was observed in sella dimensions, with a smaller anterior clinoid distance found in female patients with severe malocclusion compared to males. Sella dimensions also varied across different types of severe malocclusion: patients with mandibular deviation exhibited smaller dorsum sellae height and sella depth, while patients with hypodontia showed smaller anterior clinoid distance and left sella length. CBCT holds significant importance in orthodontic clinical practice, facilitating the identification of abnormal three-dimensional parameters.

## Supplementary Information

Supplementary Material 1.

## Data Availability

Data for this study are available by contacting the corresponding author upon reasonable request.

## Abbreviations

ACD: Anterior clinoid distance
ACP: Anterior clinoid process
ALCH: Anterior left clinoid height
ANB (∠ANB): A (subspinale) point–Nasion–B (supramental) angle
ANS: Anterior nasal spine
ANS–Me: Lower facial height
ANS–PNS: Maxillary length (palatal plane)
ARCH: Anterior right clinoid height
Ba: Basion
CBCT: Cone-beam computed tomography
Co: Condylion
Co-Go: Mandibular ramus height
∠Co-Go-Me: Mandibular angle
DS: Dorsum sellae
DSH: Dorsum sellae height
FH: Frankfort horizontal
∠FH-SN: Anterior cranial base angle
FMA: Frankfort-mandibular plane angle
Gn: Gnathion
Go: Gonion
Go-Gn: Mandibular body length
ICC: Intraclass correlation coefficient
∠L1-MP: Lower incisor–mandibular plane angle
LSL: Left sella length
Me: Menton
∠MP-FH: Mandibular plane to Frankfort horizontal angle
N: Nasion
N-ANS: Upper facial height
N-Ba: Total cranial base length
N-Gn: Total facial height
N-Me: Total anterior facial height
∠NA-PA: Nasion–A point to the A-Po (pogonion) line
OBD: Open bite distance
ODI: Overbite depth indicator
Or: Orbitale
PDD: Pogonion deviation distance
PCD: Posterior clinoid distance
PCP: Posterior clinoid process
PLCH: Posterior left clinoid height
PNS: Posterior nasal spine
PRCH: Posterior right clinoid height
Po: Porion
RSL: Right sella length
S: Sella (center of sella turcica)
S-Ba: Posterior cranial base length
S-Go: Posterior facial height
S-N: Anterior cranial base length
SDe: Sella depth
SDi: Sella diameter
SL: Sella length
SN: Sella-Nasion plane
∠SN-GoGn: Sella–Nasion to gonion–gnathion angle
STB: Sella turcica bridging
TS: Tuberculum sellae
TSH: Tuberculum sellae height
∠Ba-S-N: Total cranial base angle
