# Supplementary material for: Sella turcica dimensions in patients with severe skeletal malocclusion using cone-beam computed tomography: a retrospective observational study
Source: BMC Oral Health. 2026 Aug 14;26:2228. doi: 10.1186/s12903-026-09595-7 (PMC13749651; doi:10.1186/s12903-026-09595-7)
Supplement: Supplementary file 1 [file 12903_2026_9595_MOESM1_ESM.docx]

**Supplemental Tables**

**Supplemental Table 1 The cephalometric measurements, open bite distance, pogonion deviation distance, hypodontia and age distribution of six group in genders**

|  | Normal | | Skeletal Class III | | Skeletal Class II | | Open Bite | | Mandibular Deviation | | Hypodontia | |
| --- | --- | --- | --- | --- | --- | --- | --- | --- | --- | --- | --- | --- |
|  | Male | Female | Male | Female | Male | Female | Male | Female | Male | Female | Male | Female |
| ∠ANB（°） | 3.05±  1.51 | 3.24±  1.11 | -5.05±  2.19 | -5.09±  1.69 | 8.93±  1.53 | 9.09±  1.23 | 2.23±  2.61 | 2.52±  2.86 | -0.55±  2.04 | 0.49±  2.26 | 3.60±  3.25 | 2.70±  2.65 |
| Wits(mm) | -1.04±1.69 | -0.13±1.83 | -18.46±24.89 | -12.56±3.72 | 6.90(4.00,9.40) | 5.60(4.00,7.90) | -3.19±6.09 | -0.22±4.19 | -6.11±4.75 | -6.57±3.52 | 0.39±2.79 | -1.05±3.95 |
| ∠NA-PA（°） | 7.29±  4.56 | 6.72±  3.14 | -10.68±  5.45 | -9.79±  5.57 | 19.22±  4.51 | 20.14±  3.56 | 5.59±  5.47 | 6.98±  7.63 | -1.00(-3.60,0.70) | -0.50(-3.50,5.65) | 5.86±  7.99 | 4.38±  5.98 |
| ∠L1-MP（°） | 95.97±  6.24 | 97.17±  5.23 | 78.49±  8.74 | 78.70±  8.02 | 106.85±  9.17 | 100.94±  7.65* | 90.60±  8.52 | 92.17±  8.66 | 85.68±  7.96 | 85.40±7.35 | 93.00±  10.26 | 91.71±  8.88 |
| ODI（°） | 71.04±  5.42 | 71.59±  3.78 | 53.96±  7.43 | 52.05±  7.23 | 83.65±  11.03 | 78.97±  6.38 | 62.50(57.80,69.00) | 66.20(58.90,70.65) | 83.41±  6.21 | 83.14±  5.01 | 75.93±  8.90 | 71.62±  7.73 |
| OBD（mm） | 0 | 0 | 0 | 0 | 0 | 0 | 4.10(3.90,5.60) | 4.00(3.30,4.30) | 0 | 0 | 0 | 0 |
| PDD（mm） | 0.84±  1.06 | 1.13±  1.04 | 2.26±  1.69 | 1.21±  1.63 | 1.05±  1.05 | 0.58±0.  84 | 1.07±  1.03 | 1.42±  1.59 | 7.10(5.20,10.20) | 7.10(4.95,7.95) | 1.42±  2.08 | 0.75±  1.10 |
| MT (n) | 0 | 0 | 0 | 0 | 0 | 0 | 0 | 0 | 0 | 0 | 3.50(3.00,5.75) | 5.50(3.25,7.75) |
| Age (year) | 19.50(16.75,27.25) | 23.00(18.50,27.25) | 19.00(17.75,21.00) | 20.50(17.00,25.00) | 21.00(17.00,27.00) | 23.00(18.00,27.00) | 18.00(17.00,20.00) | 20.00(18.00,24.50)* | 19.00(17.00,21.00) | 19.00(16.00,21.50) | 22.50(19.00,25.50) | 19.50(18.00,26.75) |

ODI: Overbite Depth Indicator, OBD: Open Bite Distance, PDD: Pogonion Deviation Distance, MT: missing teeth; n: number of subjects.

The *P*-value were obtained from independent samples t-tests or Mann-Whitney U tests. *P＜0.05.

**Supplemental Table 2 The mean and standard deviation of male sella turcica dimensions in six groups**

|  | Normal | Skeletal Class III | Skeletal Class II | Open Bite | Mandibular Deviation | Hypodontia | Overall Test | *P*-value |
| --- | --- | --- | --- | --- | --- | --- | --- | --- |
| ACD | 26.72±2.33 | 26.93±2.26 | 26.93±3.87 | 26.69±2.10 | 27.34±2.70 | 25.75±2.87 | F (5,91) =0.596 | 0.703 |
| PCD | 17.01(13.39, 19.66) | 16.98(15.34,19.52) | 17.27(14.65,19.27) | 17.52(15.65,19.17) | 17.44(14.80,18.79) | 18.69(14.98,20.44) | H (5) =1.792 | 0.877 |
| LSL | 7.46(6.36,9.37) | 6.66(5.53,10.08) | 6.44(4.95 ,7.32) | 8.53(6.11,11.09) | 7.63(6.18 ,10.27) | 6.57(3.81,8.76) | H (5) =6.955 | 0.224 |
| RSL | 6.68±2.09 | 6.74±2.90 | 6.79±2.26 | 6.86±2.14 | 7.10±2.82 | 6.12±2.64 | F (5,91) =0.267 | 0.930 |
| ARCH | 7.85±1.72 | 7.09±1.87 | 7.48±1.30 | 7.69±0.98 | 7.43±1.56 | 7.68±1.47 | F (5,91) =0.531 | 0.752 |
| PRCH | 8.63±1.69 | 8.00±1.77 | 8.98±1.94 | 8.12±1.52 | 8.44±1.48 | 8.83±2.21 | F (5,91) =0.768 | 0.575 |
| ALCH | 7.45±1.28 | 6.93±1.88 | 7.55±1.23 | 7.69±1.49 | 6.92±1.32 | 7.89±1.77 | F (5,91) =1.124 | 0.353 |
| PLCH | 8.58±2.19 | 7.42±1.65 | 7.59±1.95 | 7.76±2.01 | 7.95±1.39 | 8.23±2.33 | F (5,91) =0.828 | 0.533 |
| TSH | 8.08±1.34 | 7.67±1.93 | 8.29±0.80 | 8.05±0.76 | 7.69±1.59 | 8.12±1.28 | Welch's F (5, 42.003) = 0.518 | 0.761 |
| DSH | 8.17±1.75 | 6.83±1.84 | 7.69±1.82 | 7.69±1.75 | 7.11±1.64 | 7.98±1.32 | F (5,91) =1.573 | 0.175 |
| SL | 10.07±1.05 | 10.65±1.62 | 9.89±1.68 | 10.42±1.31 | 10.42±1.50 | 9.67±1.91 | Welch's F (5, 41.402) =0.791 | 0.562 |
| SDi | 10.87±1.18 | 11.16±1.25 | 10.45±1.88 | 10.92±1.31 | 10.63±1.64 | 10.85±1.01 | F (5,91) =0.520 | 0.760 |
| SDe | 8.04±1.39 | 7.02±1.55 | 8.11±0.86 | 7.88±1.13 | 7.49±1.42 | 8.19±1.09 | F (5,91) =2.153 | 0.066 |

The test statistics F, H and Welch's F are derived from the one-way ANOVA, Kruskal-Wallis test and Welch's ANOVA, respectively. The *P*-value indicates statistical significance for the corresponding test.

**Supplemental Table 3 The mean and standard deviation of female sella turcica dimensions in six groups**

|  | Normal | Skeletal Class III | Skeletal Class II | Open Bite | Mandibular Deviation | Hypodontia | Overall Test | *P*-value | Cohen's d (95% CI) |
| --- | --- | --- | --- | --- | --- | --- | --- | --- | --- |
| ACD | 25.88±2.26 | 24.72±2.37 | 25.34±2.31 | 26.19±2.31 | 24.48±2.79 | 24.04±2.54 ^a^ | F (5,137) =2.840 | 0.018 | 0.765(0.161,1.361)  Hypodontia vs Normal |
| PCD | 17.44±2.22 | 15.88±2.51 | 16.34±2.12 | 16.69±1.82 | 16.40±2.54 | 17.12±2.41 | F (5,137) =1.390 | 0.232 | 0. 657(0.460,1.260)  Skeletal Class III vs Normal |
| LSL | 6.77±1.77 | 7.29±2.60 | 6.97±1.67 | 7.95±2.29 | 7.56±2.69 | 5.95±2.08 | F (5,137) =2.392 | 0.041 | -0.573(-1.155,0.015)  Open Bite III vs Normal |
| RSL | 6.49(4.71,8.33) | 7.17(5.75,8.47) | 6.55(4.86,7.56) | 7.73(6.14,9.28) | 5.98(4.98,8.41) | 6.14(5.28,9.19) | H (5) =12.193 | 0.032 | -0.537(-1.118,0.050)  Open Bite III vs Normal |
| ARCH | 7.75±1.87 | 7.39±2.11 | 7.55±1.61 | 7.61±1.47 | 7.18±1.58 | 7.74±1.58 | F (5,137) =0.400 | 0.848 |  |
| PRCH | 8.44±1.76 | 8.27±1.91 | 8.92±2.05 | 8.60±1.45 | 7.89±1.89 | 8.82±1.34 | F (5,137) =1.140 | 0.342 |  |
| ALCH | 7.38±1.51 | 7.25±2.19 | 7.04±2.06 | 7.85±1.54 | 7.11±1.85 | 7.59±1.77 | F (5,137) =0.686 | 0.635 |  |
| PLCH | 7.89±2.26 | 7.52±2.05 | 8.12±2.23 | 8.52±1.88 | 7.93±1.87 | 8.21±1.34 | F (5,137) =0.698 | 0.626 |  |
| TSH | 8.89(7.28,10.18) | 8.51(7.43,9.08) | 8.00(6.35,9.44) | 8.83(8.07,9.47) | 7.98(7.51,9.51) | 8.15(7.30,9.57) | H (5) =4.179 | 0.524 |  |
| DSH | 8.04±1.64 | 7.92±2.14 | 8.55±1.84 | 8.00±1.45 | 7.12±1.91 | 7.95±1.50 | F (5,137) =1.719 | 0.134 |  |
| SL | 10.60(9.41,11.41) | 11.33(10.30,12.13) | 10.00(8.72,10.99) | 10.38(8.94,10.95) | 10.72(9.23,11.39) | 9.89(9.16,10.59) | H (5) =11.616 | 0.040 |  |
| SDi | 11.73±1.25 | 11.62±1.87 | 11.08±1.74 | 11.29±1.37 | 11.27±1.22 | 11.40±1.35 | F (5,137) =0.614 | 0.689 |  |
| SDe | 8.44±1.47 | 8.05±1.61 | 8.31±1.59 | 8.31±1.06 | 7.50±1.16 | 8.23±1.23 | F (5,137) =1.509 | 0.191 | 0.717(0.121,1.325)  Mandibular Deviation  vs Normal |

The test statistics F and H are derived from the one-way ANOVA and Kruskal-Wallis test. The *P*-value indicates statistical significance for the corresponding test. Significant difference vs. normal (P < 0.05) by: a, Dunnett's test; b, Bonferroni test. The a, b＜0.05；aa, bb＜0.01；aaa, bbb＜0.001. The Cohen's d (95% CI) with medium or strong effect size is provided.
